# Introductory Lecture to the Course of Therapeutics

**Published:** 1883-01

**Authors:** 


### Selections.

### INTRODUCTORY LECTURE TO THE COURSE OF THERAPEUTICS.

Delivered in the Hospital St. Antoine by Prof. Dujardin-Beaumetz, of Paris.

*Gentlemen:* I owe you an explanation of the words "Clinical Therapeutics" placed at the head of these lectures. What is meant by Clinical Therapeutics? What are the limits? Deriving the name as we do from two distinct departments of medical study, what part shall we assign to therapeutical, what part to clinical medicine? On all these points I will endeavor to be explicit.

*Clinical Medicine and Clinical Therapeutics.*—When you study therapeutics, so-called, you pass in review the different medicaments which constitute the materia medica; you learn their natural history, their physiological properties, their dosage and their various applications in the treatment of diseases.

This method of study is altogether theoretical. It is like commencing clinical medicine by learning about diseases, their march and their symptoms in treatises on Pathology and Practice; but in order that Therapeutics, like Pathology, may become practical, useful, productive, the theoretical notions which you have learned must be applied to the patient; moreover, as Clinical Medicine is the study of the modifications which different organisms impose on the course of morbid affections, so clinical therapeutics will enable you to appreciate in the living being the constantly occuring deviations from the precise laws formulated by therapeutics properly so-called.

To examine and survey the effects of different remedial agents administered to the patient, and to study their indications, will constitute, for us, clinical therapeutics.

In pursuit of this knowledge you will learn not only how to handle medicines, but also how to associate them so as to constitute what as been described under the name of medication. No study more practical, none more necessary.

*Utility of Therapeutics.*—In this place to insist on the necessity of therapeutics would be absurd; medicine without therapeutics does not exist. All that you learn about medicine properly so-called, and the sciences accessory to it, has but one end and aim—to relieve and cure the patient.

When you are in presence of a sick person, after having taxed all the knowledge you possess to enable you to arrive at an exact diagnosis, and after having carefully considered the prognosis, you are inevitably and fatally brought to face the question which your own conscience, your patient and those around him force upon you. What must be done? You will have to look to therapeutics for an answer, and the world will form their opinion of you much more from the skill with which you combat disease than from the knowledge, however pretentious, which will enable you to recognize and diagnosticate disease.

Far from our thought the intimation that one can be a good practitioner without a thorough understanding of clinical medicine and pathology; in order to institute a treatment and determine the indications, it is absolutely necessary to know (as far as possible) the symptoms and natural tendencies of the malady which is before you. In fact, everything in therapeutics will be hesitating, mismanaged, incoherent, unless you begin by establishing the treatment on a solid basis, which is an exact knoweldge of the morbid affection.

*Scepticism and Enthusiasm in Therapeutics.*—When occupied with therapeutics there are two dangerous rocks to shun—scepticism on the one hand, exaggerated enthusiasm on the other. To believe too much and not to believe at all are two opposite terms, but they are not so far apart as one might suppose. The one engenders the other, and extreme credulity gives rise to incredulity.

Beware especially of scepticism. A physician who lacks faith in medicinal measures has no more reason for existence than a priest who does not believe the religion he teaches, or a soldier destitute of love of his country and his flag. It is repugnant to reason and to conscience that he can be a good physician who judges of no utility all the remedial agents that have the sanction of tradition and custom.

*Illusions in Therapeutics.*—But, on the other hand, it often happens that he who has the reputation of being sceptical at the hospital becomes an over-zealous prescriber when at the bedside of his private patient. Believe then in your art, but that this belief may be judicious, reasonable, let it not suffer you to be too easily carried away by what you may deem the results of your medication; in therapeutics illusions are indeed very frequent. This arises from numerous causes, especially from the propensity of the human mind to attribute all that eventuates favorably in the course of the disease to the medicine given, when very often it is only the natural evolution of the disease which the physician has observed.

It is particularly in epidemic complaints that great prudence and extreme reserve should be exercised before drawing conclusions. The type of epidemics is variable, and, according as this is mild or severe, therapeutical results are different. This explains to you why it has happened that certain remedies exhibited with success in some epidemic and contagious diseases, in other seasons have failed to give as good results. This is an example of those therapeutic illusions which have incumbered the materia medica with so many drugs which have obtained a certain brief reputation in their day, soon to fall into forgetfulness and neglect until another experimenter, repeating the experiences of a bygone time, restores them to passing notoriety.

This celebrity and this decadence of remedial agents are unfortunately facts of too great frequence in therapeutics. So, after having pruned away all the useless and superfluous substances of the materia medica, if you retain only those which medical practice has consecrated by long usage, you will find that the really useful medicaments are much less numerous than one would suppose, and that your daily practice will include but a few drugs.

*Is Medicine an Art or a Science?*—For a long time the question has been discussed, "is medicine an art or a science?" It is both.

Medicine is a science by the many kinds of knowledge which it includes as a necessary part; it is art by its application to the patient, and above all by its therapeutics. It is in this art that all the talent of the physician finds its proper exercise; it is by the form given to his preparations, by a happy choice of remedial agents, by their favorable combination, that the physician is a veritable artist. And when Trousseau pronounced these words he was himself the living personification of the fact, for no one ever carried farther than he the art of therapeutics.

*Empiricism.*—Follow no exclusive method ; draw from all sources. Be not too solicitous for physiological explanations ; do not demand for each drug an absolute experimentation which may explain its therapeutical action. Because you know not the mode of action of quinine, do you any the less believe in its efficacy in intermittent fever? Because you are ignorant how mercury acts, does it cure syphilis any the less ?

I am aware that by so speaking I lay myself open to the charge of gross empiricism. It will be said, too, that I am diverting therapeutical practice from the new and scientific road which it ought to travel. But this road is scarcely marked out ; only here and there a few stakes have been set, and unhappily these stakes are not very firmly placed.

*Experimental Therapeutics.* — Experimental therapeutics, in fact, may be said to exist only in name. Being unable to induce in animals artificial diseases, we cannot study on them the therapeutical action of drugs. We have scarcely been able to arrive at a knowledge of their physiological action ; for we are too often obliged, in order to obtain appreciable effects, to produce grave disorders and to administer the drug in the state of poison rather than in the state of medicine. By this method, then, of studying the action of drugs, we have cultivated an experimental toxicology rather than experimental therapeutics. Do not think, however, that I would discourage these researches. You know, on the contrary, how much I prize them. You have often seen me in our laboratory study the effects of medicines on animals ; you have seen me examine attentively the symptoms produced. It is indeed an excellent study which has furnished valuable data, but do not forget that it is only a complementary study.

It enables us to give a tolerably plausible explanation of the action of the medicament, and especially to know the limits beyond which it is not safe to go, and at what moment the drug ceases to become medicinal and becomes a poison. But it is not physiological experimentation that decides the destination of the medicament or of medication ; it is the effect of the remedy on the sick man and on the march of the disease which determines its therapeutical value.

The history of therapeutics these last few years shows that it is by this mode of procedure that progress in this science has been made. Do you believe that it was as a sequence of experimentation on animals that chloral, bromide of potassium, alcohol, etc., were introduced into therapeutics ? No, the clinician first noted with care the favorable results obtained in the treatment of active affections, then the facts reported were confirmed by the experience of others, and the experimenter, applying the drug in his turn to animals, studied the intimate mechanism and physiological action.

It is, then, always to observation that you should have recourse ; it is to the attentive examination of the patient that you should always return. Careful observation will enable you to study the action of the drug, to lessen or modify the dose according to the indications, and to decide the proper form for administration.

*Complexity in Therapeutics.*—Do not employ too many remedies at the same time ; do not in your busy endeavors to serve your patient inflict upon him medicines and medicinal measures widely differing in their action. Study with care the disease which is before you ; go back to the origin of the morbid affection ; formulate the leading indications resulting therefrom ; decide concerning the diatheses which have influenced the course of the malady, institute a plan of treatment and endeavor to carry it out with a very moderate exhibition of drugs.

*Therapeutics of Symptoms.*—During the years just passed, we have seen it gravely urged that we should treat diseases by meeting particular symptoms ; that is to say, by combating each of the morbid phenomena by a specific medication. This is, I believe (in very many cases at least), a pernicious course to pursue, and one for which there is little scientific justification. Instead of dispensing your medicines in this way, instead of introducing into the economy numerous substances differing in their nature, differing often in their action, adopt an opposite method—that is to say, endeavor to find the point of departure of all the manifold symptoms, and to this, as the real cause, direct your medication.

*Constancy in Therapeutics.*—Be not too changeable; do not allow yourself to drift about at the caprice of your patient, who would fain experience immediately the benefit of the medicine; learn to be patient and wait until the medicine has had time to produce all its effects. Husband well your therapeutical forces; do not expend all your efforts at once; follow the tactics of the army general, and to decide the victory keep always strong reserves.

*Coolness and Presence of Mind in Therapeutics.*—Unhappily the physician in certain cases called cases of urgency often yields to the importunities of the family, who are frightened at the progress of the disease, and administers with lavish hand medicines that are heterogeneous and even incompatible.

*Accumulation of Doses.*—In the midst of the general disorder be calm and cool; be not precipitate in the administration of remedies; act rapidly and with energy, but go right to the end you have in view, without stopping to meet secondary symptoms.

Do not forget, especially if you institute a course of treatment that must be continued for some time, that a great many remedial substances, when given for a good while, either lose their effect or produce cumulative effects in the economy. You must in these cases know how to suspend and interrupt the administration of the drug at the proper time; you must know also how to vary its administration, in order that the patient may not be disgusted with the remedy from long taking it. Remember, also, that the effects of the same medicines differ according as they are taken in massive or fractional doses.

*The Art of Prescribing.*—This is not all; it is desirable that the physician should use the utmost care in prescribing his medicines. The hospital practice does not, unfortunately, favor this special study; we find ourselves in a particular situation which obliges us to formulate too rapidly and incompletely, so that after having followed for several years our hospital services the most of you are almost entirely ignorant of the art of prescribing.

This ignorance has more serious consequences than you think of, and if we see in our day pharmaceutical specialties having a constantly increasing importance, it is in some measure due to the fact that physicians do not acquire that expertness in the preparation of their medicines which they ought to possess, and prefer lazily to rely on the combinations of the manufacturing chemist, or even on the so-called "quack medicines."

But if by pursuing this course the practitioner often promotes the fortune of the pharmacist, he despoils himself in the end, for the patient, beguiled by the advertisements which accompany his nostrum, is almost certain to apply in the future, not to his physician, but to the vender of the patent medicine.

Learn, then, skilfully to prescribe, and not only to write in an orderly and judicious manner the substances which compose your prescription, but also to render the combination as pleasant to the taste as possible. Repudiate, therefore, in a general way all the specialties which imitate the therapeutics of to-day. Exercise the greatest care in the directions which you give to the patient or his nurses; do not fear to enter into the minutest details; indicate how the external applications should be made, and the times for giving the internal remedy; regulate carefully the little incidents of the day, and be particular about the diet, for you must ever remember that pharmaceutical measures go but a little way in the cure of your patient, and that you can often accomplish more by hygiene than you can by medicine.

*Hygiene in Therapeutics.*—Hygiene is, in fact, called upon to play a preponderating part in the treatment of diseases, and especially of chronic affections. To establish with care and in a scientific manner the bases of dietetics, ought to be one of the most serious occupations of the practitioner, and you will see in the course of these lectures the prominence which I give to hygiene in the treatment of diseases.

*Etiology in Therapeutics.*—By the side of hygienic therapeutics it is also necessary to bring into light the importance of a study of the causes of the disease, for the old adage, *sublata causa tollitur effectus*, is always true. Therefore Prof. Bouchardat was right in insisting that etiology is as indispensable to therapeutics as is hygiene, or the administration of medicines.

Pardon me, gentlemen, for these remarks, but in undertaking the responsibilities of private practice you will soon learn how all these details contribute toward the reputation which the physician enjoys. The patient cannot, in fact, judge of your technical knowledge; he appreciates simply the care which you give him, the devotion and skill which you display in such cases; he forms his opinion of you and values you by the little details of our art. Do not think lightly then of these details, to which you will see me again and again return in the treatment of our patients.

